# *Bacteroides thetaiotaomicron*-derived outer membrane vesicles promote regulatory dendritic cell responses in health but not in inflammatory bowel disease

**DOI:** 10.1186/s40168-020-00868-z

**Published:** 2020-06-08

**Authors:** Lydia Durant, Régis Stentz, Alistair Noble, Johanne Brooks, Nadezhda Gicheva, Durga Reddi, Matthew J. O’Connor, Lesley Hoyles, Anne L. McCartney, Ripple Man, E. Tobias Pring, Stella Dilke, Philip Hendy, Jonathan P. Segal, Dennis N. F. Lim, Ravi Misra, Ailsa L. Hart, Naila Arebi, Simon R. Carding, Stella C. Knight

**Affiliations:** 1 Antigen Presentation Research Group, Imperial College London, Northwick Park & St. Mark’s Hospital Campus, Watford Rd, Harrow, Greater London HA1 3UJ UK; 2 Gut Microbes and Health Research Programme, Quadram Institute Bioscience, Norwich, NR4 7UQ UK; 3 Norwich Medical School, University of East Anglia, Norwich, NR4 7TJ UK; 4 Department of Biosciences, Nottingham Trent University, Clifton Campus, Nottingham, NG11 8NS UK; 5 Food Microbial Sciences Unit, University of Reading, Whiteknights, Reading, RG6 6UR UK; 6 St Mark’s Hospital, London North West University Healthcare NHS Trust, Harrow, Greater London HA1 3UJ UK

**Keywords:** Dendritic cells, Outer membrane vesicles, *Bacteroides thetaiotaomicron*, Interleukin-10, Inflammatory bowel disease

## Abstract

**Background:**

*Bacteroides thetaiotaomicron* (Bt) is a prominent member of the human intestinal microbiota that, like all gram-negative bacteria, naturally generates nanosized outer membrane vesicles (OMVs) which bud off from the cell surface. Importantly, OMVs can cross the intestinal epithelial barrier to mediate microbe-host cell crosstalk involving both epithelial and immune cells to help maintain intestinal homeostasis. Here, we have examined the interaction between Bt OMVs and blood or colonic mucosa-derived dendritic cells (DC) from healthy individuals and patients with Crohn’s disease (CD) or ulcerative colitis (UC).

**Results:**

In healthy individuals, Bt OMVs stimulated significant (*p* < 0.05) IL-10 expression by colonic DC, whereas in peripheral blood-derived DC they also stimulated significant (*p* < 0.001 and *p* < 0.01, respectively) expression of IL-6 and the activation marker CD80. Conversely, in UC Bt OMVs were unable to elicit IL-10 expression by colonic DC. There were also reduced numbers of CD103^+^ DC in the colon of both UC and CD patients compared to controls, supporting a loss of regulatory DC in both diseases. Furthermore, in CD and UC, Bt OMVs elicited a significantly lower proportion of DC which expressed IL-10 (*p* < 0.01 and *p* < 0.001, respectively) in blood compared to controls. These alterations in DC responses to Bt OMVs were seen in patients with inactive disease, and thus are indicative of intrinsic defects in immune responses to this commensal in inflammatory bowel disease (IBD).

**Conclusions:**

Overall, our findings suggest a key role for OMVs generated by the commensal gut bacterium Bt in directing a balanced immune response to constituents of the microbiota locally and systemically during health which is altered in IBD patients.

Video Abstract

## Introduction

The human gastrointestinal (GI) tract contains an estimated 3.8 × 10^13^ bacteria (10^11^/mL contents) that play an essential role in digestion, pathogen resistance and the development of different sensory systems within the GI tract including the immune system [1–3]. Gram-negative *Bacteroides thetaiotaomicron* (Bt) is a prominent member of the intestinal microbiota of many animals [4–6]. Bt has important functions in nutrient absorption and promoting barrier function via its effect on goblet cell development and mucus secretion [7, 8]. In addition*,* Bt has been described to be both protective and pathogenic in rodent models of intestinal inflammation [9–11]. Typical of all gram-negative bacteria, Bt generates nanosized outer membrane vesicles (OMVs) during its normal growth [12, 13]. The content of OMVs is varied, including enzymes and hydrolases, cell-wall components such as lipooligosaccharide and peptidoglycan, nucleic acids and metabolites [14–16]. Importantly, OMVs are packaged within a lipid bilayer which protects them from physical, chemical and biological degradation within the GI tract [17, 18].

The production of OMVs by pathogenic gram-negative bacteria including *Vibrio cholerae*, *Neisseria meningitidis*, *Helicobacter pylori* and *Haemophilus influenzae* plays a central role in infection and the delivery of toxins to host cells, and stimulation of the immune system [13, 19–22]. OMVs produced by commensal bacterial species of the genus *Bacteroides* have been implicated in microbial and immune homeostasis. For example, polysaccharide A expressed on the surface of OMVs from *Bacteroides fragilis* can promote both regulatory T cell responses and production of interleukin (IL)-10 by dendritic cells (DC) in a TLR-2 and Gadd45α-dependent manner that contributes to protection in a mouse model of acute colitis [23]. Bt produces OMVs containing phosphatases and sulfatases which are implicated in epithelial intracellular signalling and immunomodulatory functions [24, 25]. In fact, OMVs from both pathogenic and commensal strains have been used in vaccine formulations, supporting their role in microbe-host immune system crosstalk [26–29].

Most of our understanding of OMVs-host interactions comes from animal model systems with the human immune response to Bt and OMVs being poorly characterised. Therefore, we have examined how DC from healthy individuals respond to Bt OMVs and how these responses change in individuals with inflammatory bowel disease (IBD)*.* The IBDs, including Crohn’s disease (CD) and ulcerative colitis (UC), are chronic, relapsing-remitting diseases of the GI tract affecting more than 0.3% of western populations [30]. Both CD and UC are characterised by destruction of the mucosal barrier leading to inappropriate responses to the microbiota. These altered responses stem from changes in the underlying immune system, including loss of key populations of regulatory innate and adaptive immune cells leading to overexuberant pathogenic T cell responses [31–36]. These changes are also associated with intestinal microbial dysbiosis characterised by reductions in *Bacteroidetes* and *Firmicutes* with concomitant increase in *Proteobacteria* [37].

IL-10 is an immunoregulatory cytokine and is essential for intestinal homeostasis as demonstrated in mice lacking either IL-10 or IL-10 receptor genes that spontaneously develop inflammatory disease [38, 39]. IL-10 is important for regulating T cell responses to the commensal microbiota and largely acts via the innate immune system, including macrophages and DC [40–42]. In humans, the importance of the IL-10 pathway in regulating immune response is clear from patients with monogenic defects in *IL10* and *IL10R* genes that develop rapid onset IBD [43–45].

DC are key antigen-presenting cells that both produce and respond to IL-10 to regulate immune responses [42, 46], with both human studies and mouse models demonstrating their central role in immune homeostasis and microbial tolerance [47–50]. This, together with the alterations to DC subsets in IBD [32–34, 51, 52], led us to hypothesise that DC are key effectors in the healthy response to Bt that is mediated by OMVs and that DC responses to Bt-derived OMVs may be altered in IBD.

## Results

### Bt OMVs elicit production of immunoregulatory IL-10 by DCs of the healthy human colon

**Fig. 1:**
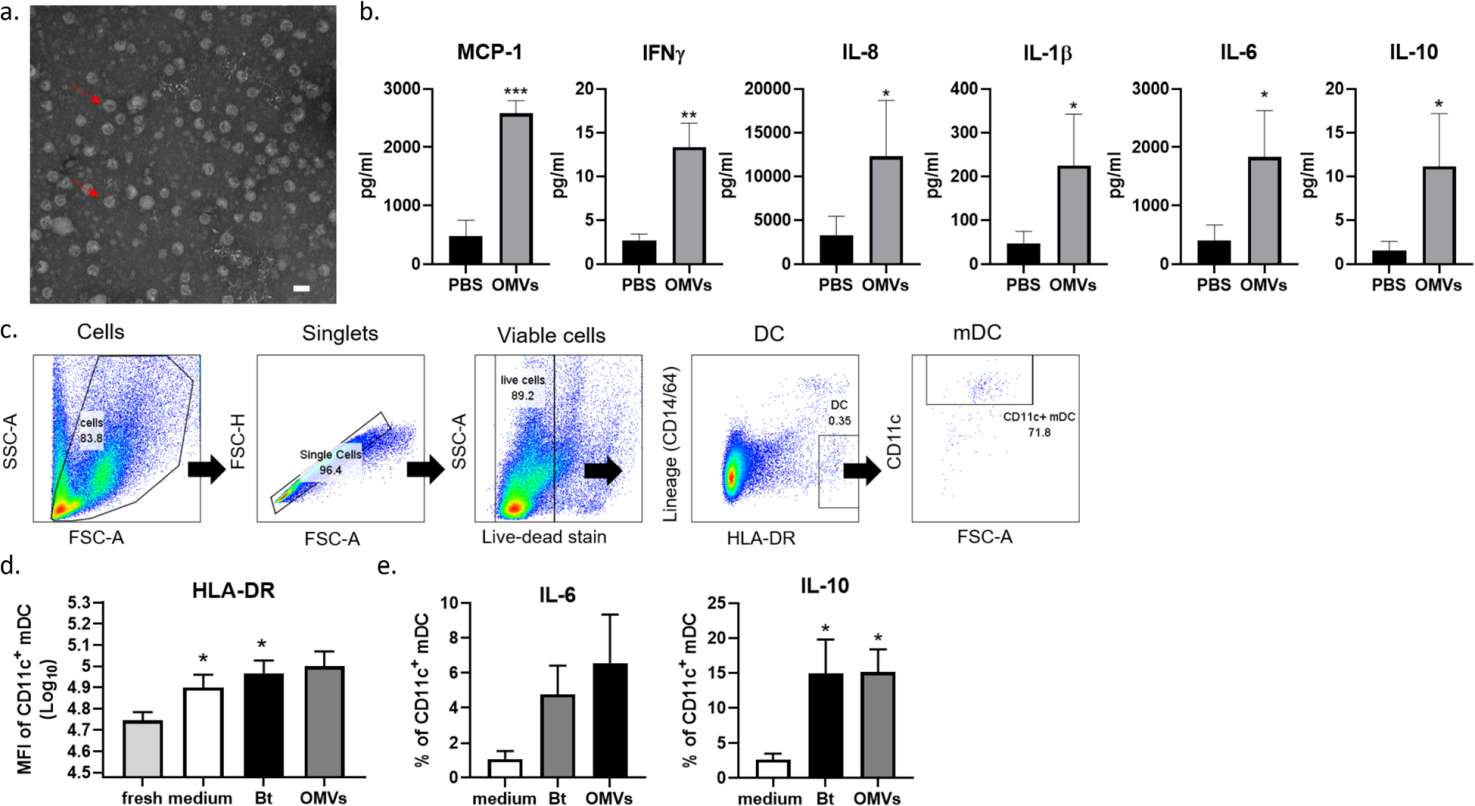
Bt OMVs stimulate production of immunoregulatory IL-10 from whole colonic biopsies and colonic LP DC. During normal anaerobic growth, Bt cells produce and release OMVs into the external milieu. **a** Electron microscope photograph of OMVs collected by sterile filtration of Bt culture supernatants through 0.22 μm membranes. Red arrows point to spherical nanostructures consisting of lipid bilayer. Scale bar, ~ 100 nm. Colonic biopsies taken from the rectosigmoid junction of healthy individuals were grown in a pIVOC system mucosal side up within Snapwell inserts for 6 h in IVOC medium with or without 10^8^-10^9^ Bt OMVs. **b** Following culture, biopsies were snap-frozen and cytokines and chemokines were measured within tissue lysates by LEGENDplex cytokine bead array. Amounts of MCP-1, IFN-γ, IL-8, IL-1β, IL-6, and IL-10 shown are mean ± SEM values from *n* = 4 healthy controls with two experimental replicates each. Statistical significance was determined using nonparametric Mann-Whitney *U* tests; *p < 0.05; **p < 0.01; ***p < 0.001. Five biopsies each from proximal and distal colon of healthy individuals were washed with DTT/EDTA and digested with collagenase/liberase to obtain total LP cells. Cells were then cultured for 20 h in the presence of either killed 10^7^ Bt/mL or 10^10^ Bt OMV/mL in complete RPMI medium, and mDC were examined by flow cytometry. **c** Identification of mDC within the total LP cells. **d** Mean fluorescence intensity of HLA-DR on total mDC in response to medium only, Bt or Bt OMVs. Statistical significance was determined by one-way ANOVA with Dunnett’s multiple comparisons test; *p < 0.05. **e** Proportion of mDC expressing IL-6 (top panel) and IL-10 (bottom panel) within the colonic LP. Pooled data from *n* = 6 HC. Statistical significance was determined using non-parametric Kruskal-Wallis ANOVA with Dunn’s multiple comparisons test; *p < 0.05

During normal anaerobic growth, Bt cells produce and release OMVs into the external milieu which consists of nanostructures exhibiting a typical spherical lipid bilayer (Fig. 1a) [53]. In order to examine mucosal immune responses to OMVs, colonic biopsies from healthy individuals (see Table 3) were cultured intact in a polarised in vitro culture system (pIVOC) for 6 h with 10^8^-10^9^ Bt OMVs/mlor medium alone added to the apical surface. Immune mediators were then measured in tissue lysates. Amongst the panel of 13 cytokines/chemokines tested (see the “Methods” section) all but IFNα2 were induced at detectable levels though TNF-α, IL-17a, IL-12p70 and IL-23 were very low (< 5 pg/ml) (Supplementary Figure 1a). Those cytokines significantly upregulated in response to OMVs were chemoattractant MCP-1 (CCL2) (*p* < 0.001), IFN-γ (*p* < 0.01), IL-8, IL-1β, IL-6 and notably IL-10 (all *p* < 0.05; Fig. 1b).

To determine if DC were a source of cytokines in the pIVOC system, total lamina propria (LP) cells were obtained from five distal and five proximal colonic biopsies by enzymatic digestion and were cultured for 20 h in the presence of either non-viable (freeze-killed) Bt (10^7^ cfu/mL), Bt OMVs (10^10^/mL) or medium only. DC was identified as HLA-DR^+^ cells which did not express CD14 or CD64, markers of macrophages or monocytes (Fig. 1c). The majority (> 60%) of DC expressed CD11c, a marker of myeloid (m)DC and were activated by both Bt and its OMVs as shown by increased expression of HLA-DR (Fig. 1d). Further, while a proportion of LP mDC expressed IL-6 (5-10%) in response to Bt and OMVs, a greater and significant (*p* < 0.05) proportion of mDC expressed IL-10 in response to either Bt or Bt OMVs (10-30%) compared to medium-only control cultures (Fig. 1e), consistent with DC being a source of IL-10 detected in intact colonic biopsies (Fig. 1b). Importantly, absolute numbers of mDC expressing IL-10, but not IL-6, were also significantly increased (*p* < 0.05) after culture with Bt or Bt OMVs compared to the medium-only condition (Supplementary Figure 1b). Thus, in health, IL-10 is a predominant cytokine produced locally by DC in the colon in response to Bt and Bt OMVs.

### Bt and Bt OMVs promote protective circulating DC responses

**Fig. 2:**
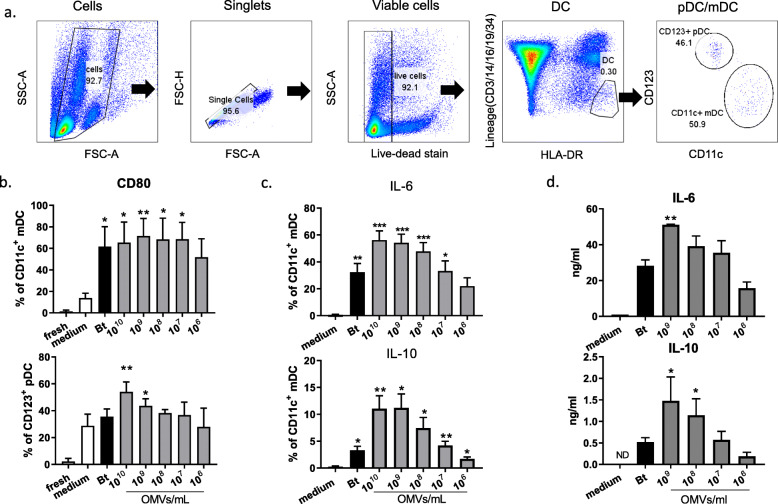
Bt OMVs activate and stimulate both IL-6 and IL-10 expression from healthy circulating DC. PBMC were prepared from healthy individuals. **a** Following 20 h culture in the presence of Bt or varying concentrations of Bt OMVs, DC were identified within live cells as HLA-DR^+^ Lineage (CD3/14/16/19/34)^−^ cells and further subdivided into CD11c^+^ myeloid DC or CD123^+^ pDC. **b** Pooled data (*n* = 4) showing proportion of mDC (top panel) and pDC (bottom panel) expressing activation marker CD80. Statistical significance was determined by ordinary one-way ANOVA with Dunnett’s multiple comparisons test; *p < 0.05; **p < 0.01. **c** Intracellular cytokine expression by mDC in response to Bt and OMVs is shown as a proportion of mDC expressing IL-6 (top panel) or IL-10 (bottom panel). Statistical significance was determined by one-way ANOVA with Brown-Forsyth and Welch corrections for unequal variance and with Dunnett’s T3 multiple comparisons tests; *p < 0.05; **p < 0.01; ***p < 0.001. **d** Total amounts of secreted IL-6 (top panel) and IL-10 (bottom panel) were measured by ELISA in culture supernatants taken at 20 h. Data shown is pooled from *n* = 3-4 HC. Statistical significance was determined by ordinary one-way ANOVA with Dunnett’s multiple comparisons test (IL-10) or non-parametric Kruskal-Wallis ANOVA with Dunn’s multiple comparisons test (IL-6); *p < 0.05, **p < 0.01

To determine whether Bt and Bt OMVs elicited a similar response from circulating DC, peripheral blood mononuclear cells (PBMC) from healthy donors were stimulated with Bt or varying doses of Bt OMVs for 20 h and DC responses were examined by multiparameter flow cytometry. DC were identified within PBMC based upon the absence of expression of lineage-specific markers (CD3/CD14/CD16/CD19/CD34) and being HLA-DR^+^. Within this population, both CD11c^+^ mDC and CD123^+^ plasmacytoid (p)DC were identified with mDC predominating (Fig. 2a).

To examine how DC responded to culture with Bt and Bt OMVs, expression of the activation marker CD80 and intracellular cytokines IL-6 and IL-10 were measured by flow cytometry. Results are displayed as percentages of total mDC and pDC staining positively for these markers. Both Bt and Bt OMVs activated mDC and pDC as seen by expression of co-stimulatory marker CD80 on the majority of mDC (60-80%) and a major proportion of pDC (40-50%) (Fig. 2b). However, cytokine responses were predominated by mDC with 30-60 % expressing IL-6 in response to Bt and to a range of concentrations of Bt OMVs (Fig. 2c) while only small proportion of pDC (< 10%) produced IL-6 in response to high concentrations of Bt OMVs (Supplementary Figure 2a and b). Importantly, IL-10 was expressed by a significant (*p* < 0.01) proportion of mDC (10-15%) in response to both Bt and a range of Bt OMV concentrations but not by pDC (Fig. 2c and Supplementary Figure 2a and b). When levels of cytokines were measured in culture supernatants, there was compared to control cultures a significant (*p* < 0.01) amount of IL-6 in response to the highest concentration of Bt OMVs (10^9^/mL), while IL-10 was detected in response to Bt OMVs at concentrations of ≥ 10^8^/mL (*p* < 0.05) (Fig. 2d). These findings suggest that, as seen with mucosal DC, circulating immune responses to Bt OMVs in healthy individuals involve a balance of protective IL-6 and regulatory IL-10.

### Reduced IL-10 response to Bt OMVs and associated loss of CD103^+^ DC in IBD

CD and UC are characterised by a loss of mucosal barrier integrity, leading to translocation of bacteria and triggering of inappropriate immune responses. Alterations in colonic DC populations, including loss of regulatory CD103^+^ DC, have been demonstrated in IBD [33, 34, 49, 50]. We confirmed these findings in our patient cohort (see Table 1) by characterising mDC in LP cells from proximal and distal colon of healthy controls (HC) and individuals with CD and UC, including expression of CD103 and SIRPα (a myeloid marker) which subdivides mDC into SIRPα^+^ CD103^−^ (similar to monocyte-derived DC), SIRPα^+^ CD103^+^ (similar to CD1c^+^ cDC2) and SIRPα^−^CD103^+^ (similar to CD141^+^cDC1) DC [54–56]. Individuals with UC and CD were subdivided into active (red symbols) or inactive (black symbols) disease based on histological findings (Table 1) and blood or faecal markers of inflammation (Table 2). Inactive disease was defined as having macroscopically normal mucosa from rectum to terminal ileum (TI) from colonoscopy and/or not having inflammatory markers in blood and faeces at time of sampling. Absolute numbers of mDC were unchanged in IBD compared to HC, regardless of disease activity (Supplementary Figure 3a). Total CD103^+^ mDC were also only significantly reduced (*p* < 0.05) in the proximal colon of CD patients (Supplementary Figure 3b) and specifically those with active disease trended towards significance (*p* = 0.0562) Further, when CD103^+^ mDC were subdivided based on expression of SIRPα, both SIRPα^+^ CD103^+^ and SIRPα^−^CD103^+^ DC were significantly reduced (*p* < 0.05; *p* < 0.05) in the proximal colon of all CD patients, along with SIRPα^−^CD103^+^DC being substantially reduced (*p* = 0.0512) in the distal colon of all UC patients compared to HC (Supplementary Figure 3c, top and middle panels). However, when UC and CD patients were subdivided based on disease activity, only CD patients with active disease had substantial reductions (*p* = 0.0885) in SIRPα^+^ CD103^+^ DC and significant reductions (*p* < 0.05) in SIRPα^−^CD103^+^ DC. The numbers of SIRPα^+^ CD103^−^ DC in either the proximal or distal colon of UC and CD patients were not different to HC (Supplementary Figure 3c; bottom panels) and this held true when patients were subdivided based on disease activity. Thus, both CD and UC are characterised by loss of CD103^+^ DC in the colonic LP and in CD the loss of CD103^+^ DC occurred in individuals with active disease.

**Table 1:** Clinical characteristics of St Mark’s Hospital colonoscopy patients donating colonic biopsies

Characteristic	Healthy control (HC)	Ulcerative colitis (UC)	Crohn’s disease (CD)
*n*	27	14	10
Male/female	11/16	7/7	3/7
Age at sampling	51 (20-77)	47.3 (24-70)	47.2 (21-77)
Inflammation categories			
None		4	4
Mayo 1 (mild disease)		5	3
Mayo 2 (moderate disease)		5	1
Mayo 3 (severe disease)		0	1
Perianal disease		0	0
Chronic obstruction		0	0
Terminal ileum resection		0	1
IBD medications at sampling			
None		8	5
Aminosalicylates		5	3
Azathioprine/6-mercaptopurine		1	3
Buscopan		0	1
Non-IBD medications at sampling			
Ondansetron		0	1
None		14	9

Demographic and clinical data analysed in Figs. 1, 2, 3, 4 and 5 and Supplementary Figs 1-4

**Table 2:** Clinical characteristics of St Mark’s Hospital blood donors and healthy volunteers

Characteristic	Healthy control (HC)	Ulcerative colitis (UC)	Crohn’s disease (CD)
*n*	17	11	7
Male/female	7/10	4/7	2/5
Age at sampling	44.3 (17-86)	46 (26-65)	58.6 (23-80)
Inflammation categories			
CRP (> 5)		3	1
ESR (> 20)		1	0
Faecal calprotectin (> 55)		2	1
Symptoms at sampling			
Diverticular disease		1	0
Diversion colitis		0	1
Fistulating Crohn’s		0	1
IBD medications at sampling			
None		9	5
Aminosalicylates		2	1
Azathioprine/6-mercaptopurine		0	0
Adalimumab		0	1
Non-IBD medications at sampling			
Vitamins D/D3/ B12/B9		1	1
Loperamide		0	1
Alendronate		0	1
Metformin		1	0
Tamoxifen		1	0
Simvastatin		1	0
None		10	6

**Fig. 3:**
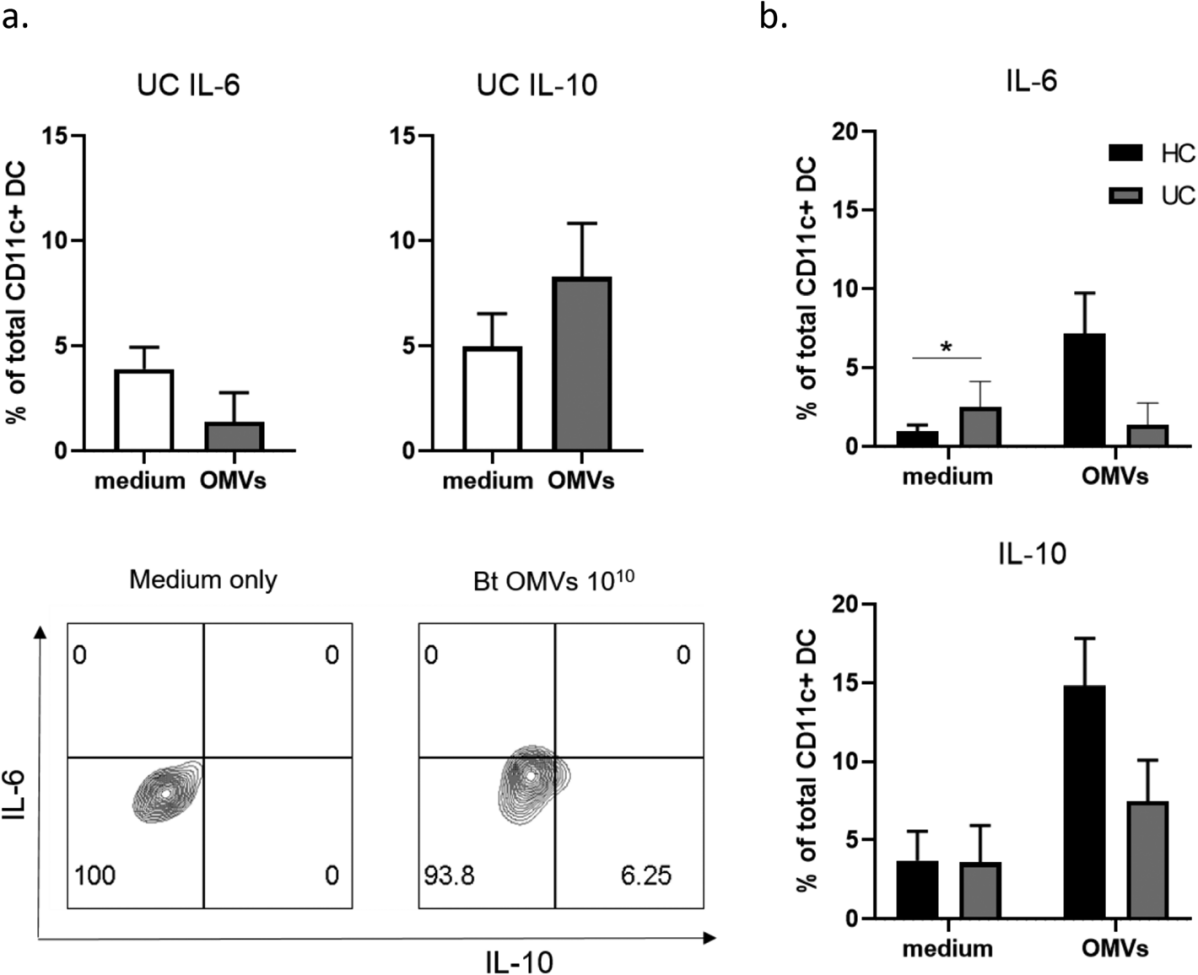
Lack of IL-10 response from colonic DC to Bt OMVs in ulcerative colitis. Following 20 h culture of LP cells from five proximal and five distal colon biopsies from UC patients with Bt OMVs 10^10^/mL or medium only, DC cytokine responses were examined by FACS. **a** Pooled data (*n* = 3) showing proportions of mDC expressing IL-6 (top left) and IL-10 (top right) and representative FACS plots from one UC patient (bottom). **b** Comparison of proportions of mDC expressing IL-6 (top) and IL-10 (bottom) in UC (*n* = 3) to HC (*n* = 6). Statistical significance was determined using unpaired *t* tests; *p < 0.05

**Fig. 4:**
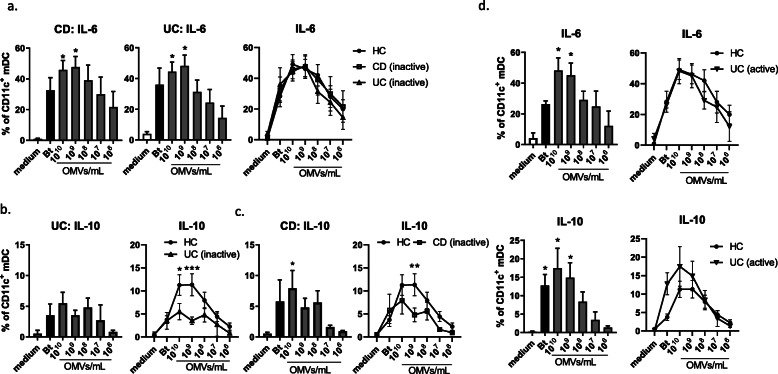
Loss of immunoregulatory IL-10 response by circulating DC to Bt OMVs in CD and UC. Whole PBMC were separated from blood of individuals with inactive Crohn’s Disease (CD) or inactive or active ulcerative colitis (UC) and intracellular cytokine analysis of mDC was examined by FACS. **a** Pooled data from inactive UC (*n* = 5) and CD (*n* = 5) patients show proportions of mDC expressing (**a**) IL-6 in response to Bt and Bt OMVs and compared to HC. **b** Pooled data from inactive UC (*n* = 5) show proportions of mDC expressing IL-10 in response to Bt and Bt OMVs and compared to HC. **c** Pooled data from inactive CD (*n* = 5) show proportions of mDC expressing IL-10 in response to Bt and Bt OMVs and compared to HC. Statistical significance was determined by Brown-Forsythe and Welch ANOVA for unequal variance with Dunnett’s T3 multiple comparisons test or two-way ANOVA with Holm Sidak’s multiple comparisons test; *p < 0.05, **p < 0.01, ***p < 0.001. **d** Pooled data from active UC (*n* = 3) showing proportions of mDC expressing IL-6 (top) or IL-10 (bottom) and comparison to HC. Statistical significance was determined by non-parametric Kruskal-Wallis ANOVA with Dunn’s multiple comparison test; *p < 0.05

**Fig. 5:**
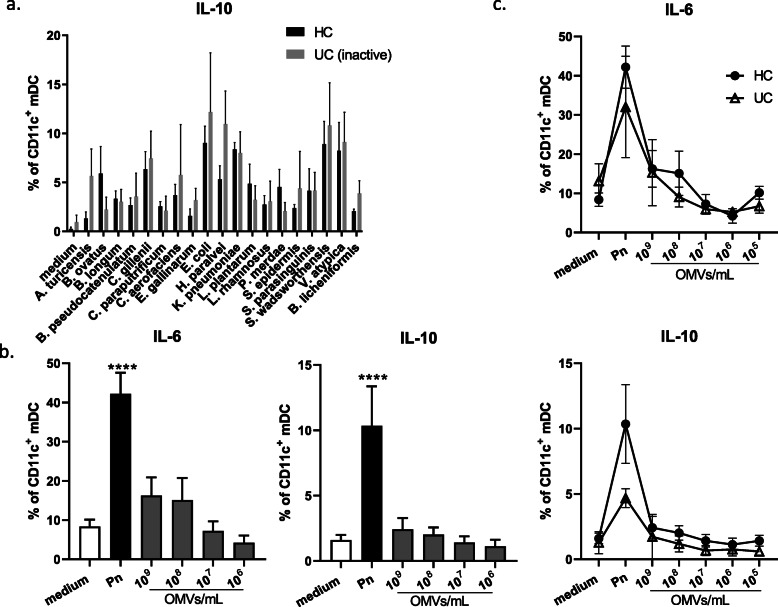
Normal IL-10 response to other commensal bacterial species or OMVs in inactive UC. Whole PBMC from healthy individuals (*n* = 4) or individuals with inactive UC (*n* = 3) were cultured for 20 h in the presence of 19 species of killed commensal bacteria. **a** Pooled data showing proportions of mDC expressing IL-10 are shown as mean (+/− SEM). PBMC from healthy individuals (HC, *n* = 4-5) or inactive UC patients (*n* = 3) were cultured for 20 h in the presence of Pn and Pn OMVs at varying concentrations. **b** The proportion of mDC expressing IL-6 or IL-10 from HC is shown. **c** Proportions of mDC expressing IL-6 (top) and IL-10 (bottom) from inactive UC and compared to HC are shown. Statistical significance was determined by ordinary one-way ANOVA with Dunnett’s multiple comparisons test or two-way ANOVA with Holm Sidak’s multiple comparisons test; ****p < 0.0001

Demographic and clinical data analysed in Figs. 1, 2, 3, 4 and 5 and Supplementary Fig 1-4

Having confirmed alterations in DC populations in IBD patients, we next investigated if the immune response to Bt OMVs was also altered in IBD patients. To this end, total LP cells from distal and proximal colon of UC patients (all with mild active (Mayo 1) disease as defined by histological findings (Table 1), and blood/faecal inflammatory markers (Table 2) were stimulated for 20 h with 10^10^ Bt OMV/mL or medium only and intracellular cytokine expression by DC was examined. Unlike in HC, Bt OMVs did not stimulate a significant proportion of mDC from LP of UC patients to express IL-6 or IL-10 (Fig. 3a). Likewise, despite similar numbers of total mDC in UC and HC, absolute numbers of IL-6- or IL-10-expressing mDC were not increased by Bt OMVs in UC (Supplementary Figure 4a and b). There was a non-significant trend towards reduced proportions and absolute numbers of mDC expressing both IL-6 and IL-10 in UC compared to HC (Fig. 3b and Supplementary Figure 4c). Thus, these findings suggest a loss of immunoregulatory DC response to Bt OMVs in UC which may in part be explained by the reduction in immunoregulatory CD103^+^ DC in these patients (Supplementary Figure 3).

### Circulating DC IL-10 response to Bt OMVs is also reduced in both UC and CD

To assess whether changes in colonic DC and response to Bt OMVs in IBD were reflected systemically in individuals with CD or UC, PBMC were used to examine circulating DC responses. In these experiments, disease activity was determined for UC and CD patients using both macroscopic histological findings from colonoscopy (Table 1) and blood or faecal markers of inflammation (Table 2). Inactive disease was defined as having macroscopically normal mucosa from rectum to terminal ileum (TI) from colonoscopy and/or not having inflammatory markers in blood and faeces at time of sampling.

As in Fig. 2a, mDC were identified in PBMC as CD11c^+^HLA-DR^+^ lineage marker (CD3/14/16/19/34) negative cells. To examine how mDC responded to culture with Bt and Bt OMVs, expression of intracellular cytokines IL-6 and IL-10 were measured by flow cytometry. Results are displayed as percentages of total mDC staining positively for these markers. Following 20 h culture, a significant proportion of mDC expressed IL-6 in response to both Bt and high doses of Bt OMVs in both inactive UC (≥ 10^9^ OMVs/mL) or inactive CD (≥ 10^9^ OMVs/mL) patients, which was comparable to the response of mDC from HC (Fig. 4a). However, neither Bt nor OMVs induced significant expression of IL-10 by mDC in inactive UC patients and the proportion of mDC that expressed IL-10 was significantly lower (*p* < 0.001; *p* < 0.05) than that of HC at high doses of OMVs (≥ 10^9^ OMVs/mL) (Fig. 4b). In patients with inactive CD, a low but significant (*p* < 0.05) proportion of mDC (10%) expressed IL-10 in response to the highest dose of OMVs (10^10^/mL) but at 10^9^/mL Bt OMVs the proportion of mDC expressing IL-10 was significantly (*p* < 0.01) reduced compared to HC (Fig. 4c). Interestingly, when DC responses to Bt and Bt OMVs were examined in UC patients with active disease, the proportion of IL-10 expressing mDC was comparable to that of healthy controls (Fig. 4d), suggesting intrinsic alterations in the immune system rather than inflammation drives the loss of regulatory response to Bt OMV in UC.

### Specificity of DC cytokine response to commensal bacteria

To determine if altered responses existed to bacteria other than Bt in individuals with UC, circulating DC responses to a selection of 19 additional human intestinal commensal bacteria were examined, including several gram-negative species. As in previous experiments, disease activity was determined for UC patients using both macroscopic histological findings from colonoscopy (Table 1) and blood or faecal markers of inflammation (Table 2). Inactive disease was defined as having macroscopically normal mucosa from rectum to TI from colonoscopy and/or not having inflammatory markers in blood and faeces at time of sampling. For UC patients with inactive disease, the proportion of mDC expressing IL-10 in PBMC in response to each of the 19 bacteria tested was comparable to HC (Fig. 5a).

We additionally examined circulating DC responses to a gram-negative commensal of the human respiratory tract, *Prevotella nanceiensis* (Pn), and its OMVs [57]. Pn, closely related to Bt, exhibits weak inflammatory properties indicating that it is tolerated by the respiratory immune system [57, 58]. In response to non-viable (freeze-killed) Pn, a proportion of mDC derived from PBMC of healthy donors expressed both IL-6 (40-50 %) and IL-10 (10%) (Fig. 5b). However, in contrast to the response to Bt OMVs (Fig. 2b), the majority (≥ 80 %) of mDC did not express either IL-6 or IL-10 in response to Pn OMVs even at the highest dose of 10^9^ OMV/mL (Fig. 5b).

Further, in inactive UC patients, the proportion of mDC expressing IL-6 or IL-10 in response to Pn OMVs was comparable to HC with only IL-10 being reduced in response to Pn (Fig. 5c). This suggests that the circulating DC IL-10 response elicited by Bt OMVs in HC may be restricted to specific members of the microbiota, with the reduced IL-10 response in inactive UC also potentially being specific to Bt OMVs.

## Discussion

Despite Bt being extensively used as a model organism to study host immune-microbiota interactions [59], the nature of the immune response to Bt in humans is poorly characterised. Here, we present novel data showing that the response of intestinal mucosal cells to Bt involves a balance in the production of protective IL-6 and regulatory IL-10 to which DC both locally and systemically contribute. Furthermore, this balanced response is mediated by OMVs, nanosized vesicles produced by the bacteria which are known to cross the mucus layer of the intestine to directly interact with immune cells beneath the boundary epithelium [60]. Furthermore, we show that in individuals with CD and UC there is a loss of regulatory IL-10 response to Bt OMVs by blood-derived DC, while protective IL-6 responses are retained. In UC, there is also a lack of colonic DC IL-10 response to Bt OMVs and this accompanies a pronounced loss of immunoregulatory CD103^+^ DC in the colonic LP. Importantly, the altered circulating DC response to Bt OMVs is only seen in UC and CD patients with inactive disease, which suggests that underlying defects in the immune system rather than inflammation drive these responses. IL-6 was significantly induced by Bt OMVs in healthy individuals both in whole colonic biopsy cultures as well as in blood DC. IL-6 is a pleiotropic cytokine produced by innate immune cells like DC in response to TLR stimulation. It has essential roles in driving B cell differentiation and antibody production as well as maintaining balanced CD4^+^ T helper responses by promoting Th17/Tfh cells and inhibiting regulatory T cells [61]. However, aberrant IL-6 responses are implicated in the pathogenesis of inflammatory diseases including Crohn’s disease and ulcerative colitis and blockade of IL-6 is protective in murine colitis [62–64]. Therefore, it is not surprising that we found an intact IL-6 response to Bt OMVs in CD and UC comparable to HC specifically in the periphery. However, in HC this was critically balanced by a significant IL-10 response which is known to directly inhibit IL-6-mediated responses [65–67]. In fact, we have shown that both CD and UC are characterised by heightened memory B cell and antibody responses to the commensal microbiota and a lack of IL-10-mediated suppression of IL-6 may directly contribute to this skewed B cell response [36]. Further, in colonic LP of UC patients, there was a clear lack of IL-6 and IL-10 production by DC to Bt OMVs suggesting a non-responsive phenotype which may contribute to a loss of colonic resident memory T cells in these patients [35, 36]. Thus, a deficient mucosal DC response to Bt OMVs, possibly due to loss of key populations of CD103^+^ DC and resident memory T cells, could lead to poorly regulated systemic responses and pathology.

The marked DC IL-10 response to Bt OMV may be specific as we did not see this response to OMVs generated by the closely related human lung commensal Pn despite a comparable IL-10 response to the whole bacterium. This may be related to regional, tissue and mucosa specific differences in addition to differences in OMV cargo and specific antigens able to elicit the IL-10 response by DC that are restricted to or are only accessible in Bt OMVs. An example of such species-specific expression of immunomodulatory molecules is the capsular polysaccharide A (PSA) antigen expressed by the closely related commensal species *Bacteroides fragilis* (Bf). The PSA antigen is expressed in Bf OMVs and elicits IL-10 production from DC in a TLR-2-dependent manner [23]. The immunomodulatory effect of Bt may also not be restricted to immune cells and DC. In the immortalised human colonic epithelial cell line Caco-2, Bt stimulates nuclear export of the NFkB subunit RelA in a mechanism involving PPARγ, which in turn dampens inflammatory responses [9]. PPARγ-mediated anti-inflammatory functions are well recognized, and agonists are being explored in the treatment of UC [68]. Bt has also been shown to contain a pyrin-like protein (PLP) which in Caco-2 cells dampens the NFkB pathways [11]. Although neither PLP nor stimulation of PPARγ has been linked to Bt OMVs directly, it is compatible with its potential immunomodulatory properties. The NFkB pathways are directly involved in transcriptional regulation of both IL-6 and IL-10 downstream of TLR stimulation [62, 67] and Bt OMVs may act via these pathways to control expression of these cytokines from DC. Future work to characterise the contents of the OMVs will be necessary to determine mechanisms of action on DC in health or IBD.

The importance of regulatory IL-10 to the healthy response of DC to Bt OMVs is congruent with observations in mice showing that Bt is colitogenic in *dnKO* mice lacking IL-10 and TGFβ-signalling pathways [10, 24]. Conversely, a strain of Bt different to that used in our study was shown to protect otherwise susceptible IL-10R-deficient mice or dextran sodium sulfate-treated mice and rats from developing colitis [11]. These discrepancies support the proposal that Bt protective functions are, at least in mice, not limited to IL-10-related pathways and may be dependent on environmental, bacterial strain- and host-specific factors [69]. Likewise in humans, Bt OMVs could elicit immunomodulatory functions via different mechanisms in addition to their role in directing balanced DC responses that could change in inflammatory settings such as in IBD. A recent study has demonstrated a key role for IL-10R signalling in DC in controlling aberrant Th1 responses both via inhibition of IL-1β and IL-12 in paediatric IBD and in an individual with *IL10RA* gene deficiency [70]. This fits with previous reports that IL-10 is both produced by and acts via DC to regulate intestinal inflammation [42]. In active UC, there was no loss of IL-10 expression by circulating mDC in response to Bt OMVs; however, it would have been of interest to additionally determine if IL-10 was able to inhibit downstream Th1 or Th17 type responses, characteristic of IBD [71, 72]. Especially given the essential role, IL-6 plays in driving inflammatory T cell responses and antibody production, Bt OMV-mediated induction of regulatory IL-10 may not be effective at dampening these responses in the context of inflammation. Likewise, it is possible that the normal IL-10 response in active UC is a result of DC or other cell types such as T or B cells having lower IL-10R expression or impaired pathway function leading to a positive feedback loop to produce more IL-10.

Unlike whole bacteria, OMVs can cross from the lumen of the intestine through the epithelial barrier and access organs and tissues beyond the GI tract [17, 60]. Exploiting this property of OMV, Bt has been successfully engineered to generate OMVs containing both therapeutic proteins and vaccine antigens for treating mucosal inflammation and generating protective mucosal and systemic immunity [28, 29]. Thus, Bt OMVs have considerable therapeutic potential. The fact that circulating DC responses to Bt and their OMVs largely reflect the local colonic DC response in health and in disease is interesting on several levels. Firstly, if intestinal disease activity can be assessed using a readout from the blood, this avoids the necessity for taking additional biopsies during colonoscopy. Secondly, the reduced IL-10 response to Bt OMVs in inactive UC was unique and could not be recapitulated in response to another related gram-negative commensal, Pn. Thus, Bt OMVs might be of use in highlighting an immune signature specific to UC that is detectable in blood samples. Finally, active inflammation appears to mask the underlying defects in DC interplay with Bt OMVs, leading to a health-associated circulating DC response. However, we did not fully explore all possible cytokines or pathways elicited by Bt OMVs and it is possible that in active IBD the signature may be distinct and different from health. For instance, Bt has been shown to elicit IL-8 from paediatric CD biopsies but not from healthy controls [73]. Additionally, reduced *IL10RA* expression and reduced responsiveness occur in a subgroup of more severe paediatric IBD patients and these are associated with enhanced IL-1β expression [70]. It will in future studies be important to identify the specific factor or factors present in Bt OMVs that trigger a healthy immune response to realise their potential use as a novel therapy for IBD.

## Conclusions

Homeostatic and regulatory immune responses are generated to commensal bacteria both locally and systemically. For Bt, these responses involve a balance of host protective IL-6 and regulatory IL-10 produced by DC. Importantly, OMVs produced by Bt are instrumental in eliciting this appropriate response both in the colonic mucosa and in the blood. In patients with UC and CD, there is a loss of regulatory IL-10 response by DC to Bt OMVs which may contribute to the inflammatory milieu of the intestine and systemically in these diseases.

## Methods

### Study design and sampling

The aim of this study was to characterise the human DC response to Bt OMVs and determine whether it is altered in IBD. Healthy donors (age 17-86) undergoing investigative colonoscopy and individuals diagnosed with CD (age 21-80) and UC (age 24-70) were recruited from outpatient clinics at St Marks Hospital, London North West University Healthcare NHS Trust. Clinical characteristics of patients and controls are in Tables 1 and 2. Active disease for CD and UC was defined as having macroscopic inflammation (rated from mild (1) to severe (3); see Table 1) from colonoscopy findings and/or presence of blood inflammatory markers C-reactive protein (CRP) > 5 and erythrocyte sedimentation rate (ESR) between 1 and 20 or faecal calprotectin > 55 (See Table 2). Inactive disease was defined as no inflammation from rectum to terminal ileum (TI) according to colonoscopy findings and/or no more than one marker of inflammation in blood (CRP > 5 or ESR between 1 and 20) and normal faecal calprotectin (< 55).

Patients were recruited over a fixed period determined by ethical permission, and no data were excluded at the end of the study. Additional healthy blood volunteers were recruited from hospital staff and visitors. Ethics approval was obtained from the Health Research Authority UK and London Brent Research Ethics Committee. Written informed consent was received from participants prior to inclusion in the study.

For whole biopsy culture experiments, healthy donors were recruited from endoscopy clinics at Norfolk and Norwich University Hospital following informed consent (see Table 3 for demographics data). Ethics approval was obtained from the University of East Anglia Faculty of Medicine and Heath Sciences Ethics Committee and Human Tissue Act Subcommittee ref 20152016-39HT and the Norfolk and Norwich University Hospital Research and Development Committee ref 20-01-16.

**Table 3:** Clinical characteristics of Norfolk and Norwich University Hospital colonoscopy patients donating colonic biopsies

Characteristic	Healthy control (HC)
*n*	4
Male/female	3/1
Age at sampling	48.5 (39-66)

Demographic and clinical data analysed in Fig. 1b and Supplementary Fig 1a

### Preparation of bacteria stocks

*Bacteroides thetaiotaomicron* VPI-5482 (Bt) (DSMZ 2079) and *Prevotella nanciensis* (Pn) (DSMZ 19126) (both from DSMZ-German Collection of Microorganisms and Cell Cultures GmbH, Braunschweig, Germany) were grown under anaerobic conditions at 37 °C in brain heart infusion (BHI) medium (Oxoid/Thermo Fisher, Basingstoke, UK) supplemented with 0.5 mg/L haemin (Sigma-Aldrich, St Louis, MO, USA) (BHI–haemin). Aliquots (1 mL) were centrifuged (13,000 rpm for 10 min), supernatants removed and cell pellets were killed by snap-freezing before storage at −80 °C. Pellets were resuspended in phosphate-buffered saline (PBS) for use. Other commensal bacteria were isolated from the caecum of healthy donors with the exception of *Collinsella aerofaciens*, which was from faeces [74–76]. Strains were grown anaerobically in Hungate tubes containing Wilkins-Chalgren broth at 37 °C for 24 h. Cell pellets were killed by snap-freezing at −80 °C and enumerated by flow cytometry following SYBR Green staining.

### Preparation of bacterial OMVs

The isolation of bacterial vesicles was done as previously described [25]. Briefly, Bt or Pn were grown under anaerobic conditions at 37 °C in an anaerobic cabinet. Bacterial starter cultures were grown overnight in 20 mL BHI medium supplemented with 15 μM haemin (Sigma-Aldrich) (BHIH). An aliquot (0.5 mL) of the starter culture was used to inoculate 500 mL BHI supplemented with 0.75 μM haemin. Cells were harvested after 16 h at an approximate OD (600 nm) of 4.0. The cells were centrifuged at 5500×*g* for 45 min at 4 °C and the supernatants filtered through polyethersulfone (PES) membranes (0.22 μm pore size) (sartorius) to remove debris and cells. Supernatants were concentrated by ultrafiltration (100 kDa molecular weight cutoff, Vivaspin 50R, sartorius), the retentate was rinsed once with 500 mL of PBS (pH 7.4) and concentrated to 0.5 mL.

Further purification was performed by fractionation of the OMV suspension by size-exclusion chromatography using a CL2-B sepharose (Sigma-Aldrich) column (120 cm × 1 cm) in PBS. The absorbance of the fractions was measured at 280 nm and the first fractions displaying an absorbance peak were pooled and concentrated down to 1 mL with a Vivaspin 20 centrifugal concentrator (100 kDa molecular weight cutoff, sartorius) and filtered through a 0.22 μm PES membrane (sartorius). Concentration of vesicles was determined using nanoparticle tracking analysis as described previously [29]. Bt OMVs were at 10^11^ vesicles/mL of PBS and Pn OMVs were at 2 × 10^10^ vesicles/mL of PBS.

### Transmission electron microscopy

Bt OMVs were observed using negative staining with transmission electron microscopy (TEM) as previously described [53]. Briefly, isolated Bt OMVs were adsorbed to carbon–formvar-coated copper EM grids (Agar Scientific) for 1 min before wicking off with filter paper and negatively staining with 2% uranyl acetate solution (BDH) in water for 1 min. Grids were air-dried before analysis using a Tecnai G2 20 Twin TEM (FEI) at ×29,000 magnification.

### Polarised in vitro organ culture of colonic biopsies

Colonic biopsies were taken from the rectosigmoid junction (around 18 cm from rectum) of macroscopically normal patients following informed consent. The polarised in vitro culture (pIVOC) of colonic biopsies used was adapted from a previous study [77]. Briefly, five colonic biopsies were collected in IVOC medium (Dulbecco’s Modified Eagle’s Medium (Sigma-Aldrich) (45 %) and distilled water (45%) containing 0.47 g NCTC-135 (Sigma-Aldrich), 0.11 g sodium bicarbonate (Sigma-Aldrich) and 10% newborn calf serum (NCS, Sigma-Aldrich). Each biopsy was orientated with the mucosal side uppermost on a cellulose nitrate filter within a Snapwell support and mounted within a well of a six-well plate containing 3 mL of IVOC medium. Once mounted, 200 μL of IVOC medium was added apically with or without 10^8^-10^9^ Bt OMVs/ml. The plate was incubated on a rotor (12 rpm) at 37 °C for 6 h, after which the biopsy was removed intact from each support, flash-frozen in liquid nitrogen and stored at −80 °C for later protein extraction and cytokine analysis.

### Biopsy tissue lysate extraction

Biopsies were thawed on ice and a mixture of 122 μl CellLytic MT (Sigma C3228), and 3 μl protease inhibitor cocktail (Sigma P2714-1BTL) was added with 5 acid-washed glass beads (3 mm diameter). Homogenisation was performed using a MP Biomedical Fastprep-24 instrument (Fisher Scientific) at 4 m/s for 30 s. Samples were centrifuged at 10,000 rpm at 4 °C for 2 min. The tissue lysate was then transferred to a 1.5 ml pre-cooled tube and centrifuged at 10,000 rpm for 10 min at 4 °C. The supernatant was carefully removed and stored at −80 °C until use for cytokine bead array (see below).

### Isolation of colonic lamina propria cells

Five proximal and five distal colon biopsies (10 mg tissue each) were obtained from macroscopically non-lesional tissue sites at routine colonoscopy in all patients as previously described [55]. Biopsies were washed in HBSS containing 1 mM DTT and 1 mM EDTA in a shaking incubator at 37 °C for 30 min to remove the epithelial layer. Supernatants were discarded and wash was repeated for a second 30 min with HBSS/DTT/EDTA. Following discard of supernatants, biopsies were further digested in RPMI medium containing collagenase D (1 mg/mL) and Liberase TL (0.1 mg/mL) for 1 h shaking at 37 °C to release the lamina propria (LP) cells. LP cells were then filtered through a 100 μM strainer, washed with PBS and centrifuged at 600 g for 5 min before proceeding to either culture or FACS staining.

### Isolation of peripheral blood mononuclear cells

Blood obtained by venepuncture was diluted 1:1 (vol:vol) in PBS and layered over Ficoll-Paque Plus (Amersham Biosciences, Chalfont St. Giles, UK). After centrifugation at 800 g for 30 min at 18 °C, PBMC were collected at the interface. PBMC were resuspended in complete medium (Dutch modified RPMI 1640 (Sigma-Aldrich, Dorset, UK) containing 100 U/mL penicillin/streptomycin, 2 mM l-glutamine, 50 μg/mL gentamicin (Sigma-Aldrich) and 10% faetal calf serum (TCS cell works, Buckingham, UK)) for culture or in PBS for FACS analysis.

### Bacterial stimulation of blood DC or LP cells

Per condition, 5 × 10^5^ PBMC were plated in 96-well U-bottom plates. PBMC were incubated with either 10^6^/mL freeze-killed commensal bacteria or OMVs at tenfold incremental concentrations from 10^10^ to 10 OMVs/mL at 37 °C, 5% CO_2_ for 20 h. LP cells were isolated as described above and resuspended in complete medium as described for PBMC. LP cells (1-2 × 10^5^ per condition) were plated in 96-well U-bottomed plates. LP cells were incubated with either 10^7^/mL freeze-killed Bt, 10^10^/mL of Bt OMVs or complete medium only at 37 °C, 5% CO_2_ for 20 h. For intracellular cytokine responses, 2 μM monensin (Biolegend) was added to wells during incubation.

### Surface marker and intracellular cytokine profiling of DC

Following incubation, PBMC and LP cells were washed with PBS and viability was determined by labelling cells with LIVE/DEAD™ Fixable Near-IR Dead Cell Stain Kit (Thermo Fisher Scientific) according to the manufacturer’s instructions. Cells were washed with FACS Buffer (1x PBS containing 2% FCS, 1 mM ETDA and 0.02% sodium azide) and then labelled with antibodies to identify the dendritic cells (Table 4). Cells were fixed in 1% paraformaldehyde (PFA) if only surface markers were examined.

**Table 4:** Antibodies used for FACS

Antigen	Clone	Isotype	Fluorochrome	Supplier
CD3	UCHT1	IgG1,k	PE Cy5	BD Bioscience
CD14	61D3	IgG1,k	PE Cy5	BioRad-Serotec
CD14	M5E2	IgG2a,k	PerCP Cy5.5	BD Bioscience
CD14	MФP9	IgG2b,k	PECF594	BD Horizon
CD16	3G8	IgG1,k	PE Cy5	BD Bioscience
CD19	HIB19	IgG1,k	PE Cy5	BD Bioscience
CD34	581	IgG1,k	PE Cy5	BD Bioscience
CD64	10.1	IgG1,k	PerCP Cy5.5	BD Bioscience
CD64	10.1	IgG1,k	PE Cy5	Abcam
CD123	6H6	IgG1,k	PE Cy7	eBioscience
HLA-DR	G46-6	IgG2a	APC	BD Bioscience
HLA-DR	L234	IgG2a	BV421	Biolegend
HLA-DR	L234	IgG2a	BV570	Biolegend
CD11c	B-Ly6	IgG1,k	BV605	BD Bioscience
CD40	LOB7/6	IgG2a	FITC	AbD Serotec (Bio-Rad)
CD40	5C3	IgG1	BV711	BD Bioscience
CD45	H130	IgG1,k	BUV395	BD Horizon
CD80	L307.4	IgG1,k	FITC	BD Bioscience
CD80	L307.4	IgG1,k	PE	BD Bioscience
CD86	BU63	IgG1	FITC	AbD Serotec (Bio-Rad)
CD86	2331(FUN-1)	IgG1,k	AlexaFluor700	BD Pharmingen
CD103 (Integrin αE)	Ber-Act8	IgG1,k	BV421	Biolegend, BD Bioscience
IL-6	MQ2-13A5	IgG1,k Rat	FITC	Biolegend
IL-6	MQ2-13A5	IgG1,k Rat	PE	eBioscience
IL-10	JES-19F1	IgG1,k Rat	PE	Biolegend
IL-10	JES-19F1	IgG1,k Rat	APC	BD Bioscience/Biolegend
Integrin β7	FIB504	IgG2a, Rat	PE	Biolegend
Integrin β7	FIB504	IgG2a, Rat	FITC	Biolegend
Integrin β7	FIB504	IgG2a, Rat	APC	BD Bioscience
SIRPα (CD172a/b)	SE5A5	IgG1,k	PECy7	Biolegend

List of all antibodies used to identify and characterise human DC in circulation and in colon for Figs. 1, 2, 3, 4 and 5 and Supplementary Figs 1-4

For intracellular cytokine analysis, cells were fixed using Leucoperm A buffer (Bio-Rad) and permeabilised using Leucoperm B (Bio-Rad) and then labelled with antibodies to interleukin (IL)-10 and IL-6 (Table 4). Finally, cells were fixed again in 1% PFA and stored at 4 °C.

### Flow cytometry

Single-cell suspensions were acquired on the BD FACSCanto II (BD Biosciences) or the BD LSR Fortessa (BD Biosciences). Compensation was carried out on FACS Diva software using Anti-Mouse Ig, κ/Negative Control Compensation Particles Set (BD Biosciences) conjugated to antibodies used in above labelling experiments. The ArC™ Amine Reactive Compensation Bead Kit (Thermo Fisher Scientific) was used for compensation of the LIVE/DEAD™ Fixable Near-IR Dead Cell Stain according to kit instructions. Data analysis was done using the FlowJo_v.10 software.

### Cytokine bead array

The LEGENDplex Human Inflammation Panel I (Biolegend, London, UK ) was used to simultaneously quantify 13 human cytokines/chemokines (IL-1β, IFN-α2, IFN-γ, TNF-α, MCP-1 (CCL2), IL-6, IL-8 (CXCL8), IL-10, IL-12p70, IL-17A, IL-18, IL-23, and IL-33) in biopsy tissue lysates according to the manufacturer’s instructions and analysed on a BD LSR Fortessa using the PE and APC channels. Data were analysed using the LEGENDplex data analysis software (Biolegend, London, UK). All cytokines/chemokines were detectable apart from IFN-α2 which was below detection limits.

### ELISA

PBMC were cultured with Bt or Bt OMVs as described above and cell supernatants were taken at 20 h post-stimulation and stored at −80 °C. Amounts of cytokines (IL-6 and IL-10) were measured using Human DuoSet ELISA kits (R and D systems) according to the manufacturer’s instructions. Plates were read on the Tecan Infinite F50 plate reader and data were analysed using the Magellan^TM^ software (Tecan Group Ltd, Mannedorf, Switzerland).

### Statistical analysis

Statistical analysis was carried out using the GraphPad Prism software version 8. For in vitro experiments, data were analysed using nonparametric Mann-Whitney *U* tests (Fig. 1b and Supplementary Figure 1a); unpaired *t* tests (Fig. 3); ordinary one-way ANOVA with Dunnett’s multiple comparisons test (Figs. 1d and 2b, d (IL-10), Fig. 5b and Supplementary Figure 2B), one-way ANOVA with Brown-Forsyth and Welch corrections for unequal variance with Dunnett’s T3 multiple comparisons test (Figs. 2c and 4a, b) or non-parametric Kruskal-Wallis ANOVA with Dunn’s multiple comparisons test (Figs. 1e and 2d (IL-6) and Fig. 4c) when making comparisons between experimental conditions within a single group; or ordinary two-way ANOVA with Holm’s Sidak’s multiple comparisons test (Figs. 4 and 5) for making comparisons between groups for different experimental conditions. Colonic LP DC data from HC, CD and UC patients (Supplementary Figure 3) were analysed using non-parametric Kruskal-Wallis ANOVA with Dunn’s multiple comparisons test; *p < 0.05.

## Supplementary information

**Additional file 1: Supplementary Figure 1.** Bt OMVs induce diverse cytokines from whole colon tissue and marked IL-10 response from colonic LP  mDC. **Supplementary Figure 2.** Plasmacytoid DC cytokine response to Bt and Bt OMVs in healthy controls. **Supplementary Figure 3.** Loss of CD103^+^ DC in UC and CD colonic LP. **Supplementary Figure 4.** Bt OMVs do not induce IL-10-expressing mDC in colonic LP in ulcerative colitis.

## Data Availability

The datasets used and/or analysed during the current study are available from the corresponding author on reasonable request.

## Abbreviations

Bt: *Bacteroides thetaiotaomicron*
OMVs: Outer membrane vesicles
DC: Dendritic cells
CD: Crohn’s disease
UC: Ulcerative colitis
IL: Interleukin
IBD: Inflammatory bowel disease
GI: Gastrointestinal
pIVOC: Polarised *in vitro* culture system
Pn: *Prevotella nanceiensis*
HC: Healthy control
LP: Lamina propria
PBMC: Peripheral blood mononuclear cells
mDC: Myeloid dendritic cell
pDC: Plasmacytoid dendritic cell
Th: T helper
Bf: *Bacteroides fragilis*
SIRPα: Signal regulatory protein alpha
CD103: Integrin alpha E
cDC: Conventional dendritic cell
TI: Terminal ileum
PSA: Capsular polysaccharide A

## References

[1] Sender R, Fuchs S, Milo R (2016). Revised estimates for the number of human and bacteria cells in the body. PLOS Biol..

[2] Luckey TD (1972). Introduction to intestinal microecology. Am. J. Clin. Nutr..

[3] Thursby E, Juge N (2017). Introduction to the human gut microbiota. Biochem. J..

[4] Moore WE, Holdeman LV (1974). Human fecal flora: the normal flora of 20 Japanese-Hawaiians. Appl. Microbiol..

[5] Human Microbiome Consortium Project. Structure, function and diversity of the healthy human microbiome. Nature. 2012;486:207–14. 10.1038/nature11234 PMC3564958 22699609

[6] Eckburg PB (2005). Diversity of the human intestinal microbial flora. Science (80-. ).

[7] Wrzosek L (2013). Bacteroides thetaiotaomicron and *Faecalibacterium prausnitzii* influence the production of mucus glycans and the development of goblet cells in the colonic epithelium of a gnotobiotic model rodent. BMC Biol..

[8] Hooper LV (2001). Molecular analysis of commensal host-microbial relationships in the intestine. Science (80-. ).

[9] Kelly D (2004). Commensal anaerobic gut bacteria attenuate inflammation by regulating nuclear-cytoplasmic shuttling of PPAR-γ and RelA. Nat. Immunol..

[10] Bloom SM (2011). Commensal bacteroides species induce colitis in host-genotype-specific fashion in a mouse model of inflammatory bowel disease. Cell Host Microbe.

[11] Delday M, Mulder I, Logan ET, Grant G (2019). Bacteroides thetaiotaomicron ameliorates colon inflammation in preclinical models of Crohn’s disease. Inflamm. Bowel Dis..

[12] Elhenawy W, Debelyy MO, Feldman MF (2014). Preferential packing of acidic glycosidases and proteases into bacteroides outer membrane vesicles. MBio.

[13] Chatterjee SN, Das J (1967). Electron microscopic observations on the excretion of cell-wall material by *Vibrio cholerae*. J. Gen. Microbiol..

[14] Kaparakis-Liaskos M, Ferrero RL (2015). Immune modulation by bacterial outer membrane vesicles. Nat. Rev. Immunol..

[15] Bryant WA (2017). In silico analysis of the small molecule content of outer membrane vesicles produced by *Bacteroides thetaiotaomicron* indicates an extensive metabolic link between microbe and host. Front. Microbiol..

[16] Jacobson AN, Choudhury BP, Fischbach MA (2018). The biosynthesis of lipooligosaccharide from *Bacteroides thetaiotaomicron*. MBio.

[17] Stentz R, Carvalho AL, Jones EJ, Carding SR (2018). Fantastic voyage: the journey of intestinal microbiota-derived microvesicles through the body. Biochem. Soc. Trans..

[18] Alves NJ, Turner KB, Medintz IL, Walper SA (2016). Protecting enzymatic function through directed packaging into bacterial outer membrane vesicles. Sci. Rep..

[19] Devoe IW (1973). RELEASE OF ENDOTOXIN IN THE FORM OF CELL WALL BLEBS DURING IN VITRO GROWTH OF *NEISSERIA MENINGITIDIS*. J. Exp. Med..

[20] DeVoe I (1975). Pili on meningococci from primary cultures of nasopharyngeal carriers and cerebrospinal fluid of patients with acute disease. J. Exp. Med..

[21] Fiocca R (1999). Release of *Helicobacter pylori* vacuolating cytotoxin by both a specific secretion pathway and budding of outer membrane vesicles. Uptake of released toxin and vesicles by gastric epithelium. J. Pathol.

[22] Ren D (2012). Characterization of extended co-culture of non-typeable *Haemophilus influenzae* with primary human respiratory tissues. Exp. Biol. Med..

[23] Shen Y (2012). Outer membrane vesicles of a human commensal mediate immune regulation and disease protection. Cell Host Microbe.

[24] Hickey CA (2015). Colitogenic *Bacteroides thetaiotaomicron* antigens access host immune cells in a sulfatase-dependent manner via outer membrane vesicles. Cell Host Microbe.

[25] Stentz R (2014). A bacterial homolog of a eukaryotic inositol phosphate signaling enzyme mediates cross-kingdom dialog in the mammalian gut. Cell Rep..

[26] Oster P (2005). MeNZB?: a safe and highly immunogenic tailor-made vaccine against the New Zealand serogroup B disease epidemic strain. Vaccine.

[27] Gerritzen MJH, Martens DE, Wijffels RH, van der Pol L, Stork M (2017). Bioengineering bacterial outer membrane vesicles as vaccine platform. Biotechnol. Adv..

[28] Carvalho AL (2019). Use of bioengineered human commensal gut bacteria-derived microvesicles for mucosal plague vaccine delivery and immunization. Clin. Exp. Immunol..

[29] Carvalho AL (2019). Bioengineering commensal bacteria-derived outer membrane vesicles for delivery of biologics to the gastrointestinal and respiratory tract. J. Extracell. Vesicles.

[30] Ng SC (2017). Worldwide incidence and prevalence of inflammatory bowel disease in the 21st century: a systematic review of population-based studies. Lancet.

[31] Geremia A, Biancheri P, Allan P, Corazza GR, Di Sabatino A (2014). Innate and adaptive immunity in inflammatory bowel disease. Autoimmun. Rev..

[32] Mann ER (2013). Intestinal dendritic cells: their role in intestinal inflammation, manipulation by the gut microbiota and differences between mice and men. Immunol. Lett..

[33] Magnusson MK (2016). Macrophage and dendritic cell subsets in IBD: ALDH+ cells are reduced in colon tissue of patients with ulcerative colitis regardless of inflammation. Mucosal Immunol..

[34] Mann ER (2014). Human gut dendritic cells drive aberrant gut-specific T-cell responses in ulcerative colitis, characterized by increased IL-4 production and loss of IL-22 and IFNγ. Inflamm. Bowel Dis..

[35] Hegazy AN (2017). Circulating and tissue-resident CD4 + T cells with reactivity to intestinal microbiota are abundant in healthy individuals and function is altered during inflammation. Gastroenterology.

[36] Noble A, et al. Deficient resident memory T-cell and Cd8 T-cell response to commensals in inflammatory bowel disease. J. Crohn’s Colitis. 2019. 10.1093/ecco-jcc/jjz175. 10.1093/ecco-jcc/jjz175 PMC7242004 31665283

[37] Ni J, Wu GD, Albenberg L, Tomov VT (2017). Gut microbiota and IBD: causation or correlation?. Nat. Rev. Gastroenterol. Hepatol..

[38] Kühn R, Löhler J, Rennick D, Rajewsky K, Müller W (1993). Interleukin-10-deficient mice develop chronic enterocolitis. Cell.

[39] Spencer SD (1998). The orphan receptor CRF2-4 is an essential subunit of the interleukin 10 receptor. J. Exp. Med..

[40] Zigmond E (2014). Macrophage-restricted interleukin-10 receptor deficiency, but not IL-10 deficiency, causes severe spontaneous colitis. Immunity.

[41] Shouval DS (2014). Interleukin-10 receptor signaling in innate immune cells regulates mucosal immune tolerance and anti-inflammatory macrophage function. Immunity.

[42] Girard-Madoux MJH (2016). IL-10 control of CD11c + myeloid cells is essential to maintain immune homeostasis in the small and large intestine. Oncotarget.

[43] Glocker E-O (2009). Inflammatory bowel disease and mutations affecting the interleukin-10 receptor. N. Engl. J. Med..

[44] Pigneur B (2013). Phenotypic characterization of very early-onset IBD due to mutations in the IL10, IL10 receptor alpha or beta gene. Inflamm. Bowel Dis..

[45] Begue B (2011). Defective IL10 signaling defining a subgroup of patients with inflammatory bowel disease. Am. J. Gastroenterol..

[46] de Smedt T (1997). Effect of interleukin-10 on dendritic cell maturation and function. Eur. J. Immunol..

[47] Loschko J (2016). Absence of MHC class II on cDCs results in microbial-dependent intestinal inflammation. J. Exp. Med.

[48] Stagg AJ, Hart AL, Knight SC, Kamm MA (2004). Interactions between dendritic cells and bacteria in the regulation of intestinal immunity. Best Pract. Res. Clin. Gastroenterol..

[49] Coombes JL (2007). A functionally specialized population of mucosal CD103 + DCs induces Foxp3 + regulatory T cells via a TGF-β– and retinoic acid–dependent mechanism. J. Exp. Med..

[50] Sun C-M (2007). Small intestine lamina propria dendritic cells promote de novo generation of Foxp3 T reg cells via retinoic acid. J. Exp. Med..

[51] Al-Hassi HO (2014). Altered human gut dendritic cell properties in ulcerative colitis are reversed by *Lactobacillus plantarum* extracellular encrypted peptide STp. Mol. Nutr. Food Res..

[52] Mann ER (2013). Dysregulated circulating dendritic cell function in ulcerative colitis is partially restored by probiotic strain *Lactobacillus casei* Shirota. Mediators Inflamm..

[53] Stentz R (2015). Cephalosporinases associated with outer membrane vesicles released by Bacteroides spp. protect gut pathogens and commensals against β-lactam antibiotics. J. Antimicrob. Chemother.

[54] Watchmaker PB (2014). Comparative transcriptional and functional profiling defines conserved programs of intestinal DC differentiation in humans and mice. Nat. Immunol..

[55] Bernardo D (2016). Chemokine (C-C Motif) receptor 2 mediates dendritic cell recruitment to the human colon but is not responsible for differences observed in dendritic cell subsets, phenotype, and function between the proximal and distal colon. Cell. Mol. Gastroenterol. Hepatol.

[56] Mann ER (2016). Compartment-specific immunity in the human gut: properties and functions of dendritic cells in the colon versus the ileum. Gut.

[57] Larsen JM (2015). Chronic obstructive pulmonary disease and asthma-associated Proteobacteria, but not commensal Prevotella spp., promote toll-like receptor 2-independent lung inflammation and pathology. Immunology.

[58] Larsen JM (2017). The immune response to Prevotella bacteria in chronic inflammatory disease. Immunology.

[59] Xu J (2003). A genomic view of the human-*Bacteroides thetaiotaomicron* symbiosis. Science (80-. ).

[60] Jones, E. J. et al. The uptake, trafficking and biodistribution of *Bacteroides thetaiotaomicron* generated outer membrane vesicles. Front. Microbiol. In Press, (2020). 10.3389/fmicb.2020.00057 PMC7015872 32117106

[61] Tanaka, T., Narazaki, M. & Kishimoto, T. IL-6 in inflammation, immunity, and disease. Cold Spring Harb. Perspect. Biol*.* 6, a016295–a016295 (2014). 10.1101/cshperspect.a016295 PMC4176007 25190079

[62] Tanaka T, Kishimoto T (2014). The biology and medical implications of interleukin-6. Cancer Immunol. Res..

[63] Waldner MJ, Neurath MF (2014). Master regulator of intestinal disease: IL-6 in chronic inflammation and cancer development. Semin. Immunol..

[64] Bernardo D (2012). IL-6 promotes immune responses in human ulcerative colitis and induces a skin-homing phenotype in the dendritic cells and T cells they stimulate. Eur. J. Immunol..

[65] de Waal Malefyt R, Abrams J, Bennett B, Figdor CG, de Vries JE (1991). Interleukin 10 (IL-10) inhibits cytokine synthesis by human monocytes: an autoregulatory role of IL-10 produced by monocytes. J. Exp. Med..

[66] Fiorentino DF, Zlotnik A, Mosmann TR, Howard M, O’Garra A (1991). IL-10 inhibits cytokine production by activated macrophages. J. Immunol..

[67] Saraiva M, Vieira P, O’Garra A (2020). Biology and therapeutic potential of interleukin-10. J. Exp. Med..

[68] Da Silva S (2018). A novel topical PPARγ agonist induces PPARγ activity in ulcerative colitis mucosa and prevents and reverses inflammation in induced colitis models. Inflamm. Bowel Dis..

[69] Sitkin, S. & Pokrotnieks, J. Gut microbiota as a host defender and a foe: the 2 faces of commensal *Bacteroides thetaiotaomicron* in inflammatory bowel disease. Inflamm. Bowel Dis. 25, e71–e71 (2019). 10.1093/ibd/izy377 30561647

[70] Veenbergen S (2019). IL-10 signaling in dendritic cells controls IL-1β-mediated IFNγ secretion by human CD4+ T cells: relevance to inflammatory bowel disease. Mucosal Immunol..

[71] Huber S (2011). Th17 cells express interleukin-10 receptor and are controlled by Foxp3− and Foxp3+ regulatory CD4+ T cells in an interleukin-10-dependent manner. Immunity.

[72] Liu B, Tonkonogy SL, Sartor RB (2011). Antigen-presenting cell production of IL-10 inhibits T-Helper 1 and 17 cell responses and suppresses colitis in mice. Gastroenterology.

[73] Edwards LA (2011). Aberrant response to commensal *Bacteroides thetaiotaomicron* in Crohnʼs disease. Inflamm. Bowel Dis..

[74] Hoyles L (2015). Klebsiella pneumoniae subsp. pneumoniae–bacteriophage combination from the caecal effluent of a healthy woman. PeerJ.

[75] Hoyles L (2018). Metabolic retroconversion of trimethylamine N-oxide and the gut microbiota. Microbiome.

[76] Thorasin T, Hoyles L, McCartney AL (2015). Dynamics and diversity of the ‘Atopobium cluster’ in the human faecal microbiota, and phenotypic characterization of ‘Atopobium cluster’ isolates. Microbiology.

[77] Schüller S, Lucas M, Kaper JB, Girón JA, Phillips AD (2009). The ex vivo response of human intestinal mucosa to enteropathogenic *Escherichia coli* infection. Cell. Microbiol..

