# Supplementary material for: Bacteroides thetaiotaomicron-derived outer membrane vesicles promote regulatory dendritic cell responses in health but not in inflammatory bowel disease
Source: Microbiome. 2020 Jun 8;8:88. doi: 10.1186/s40168-020-00868-z (PMC7282036; doi:10.1186/s40168-020-00868-z)
Supplement: Supplementary file 2 — Additional file 1: Supplementary Figure 1. Bt OMVs induce diverse cytokines from whole colon tissue and marked IL-10 response from colonic LP mDC. Supplementary Figure 2. Plasmacytoid DC cytokine response to Bt and Bt OMVs in healthy controls. Supplementary Figure 3. Loss of CD103+ DC in UC and CD colonic LP. Supplementary Figure 4. Bt OMVs do not induce IL-10-expressing mDC in colonic LP in ulcerative colitis. [file 40168_2020_868_MOESM1_ESM.pptx]

## Slide 1
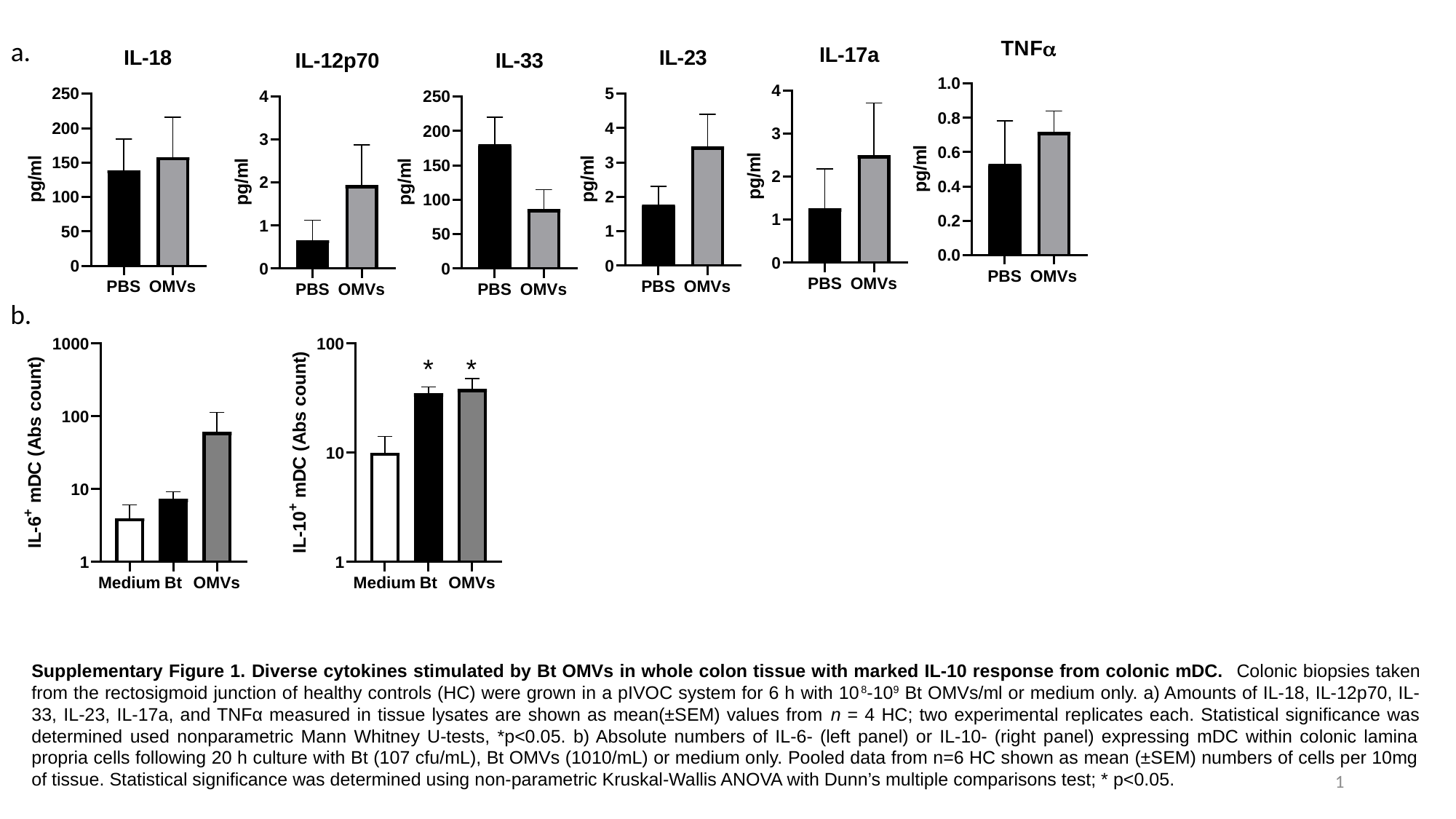

a.
b.
Supplementary Figure 1. Diverse cytokines stimulated by Bt OMVs in whole colon tissue with marked IL-10 response from colonic mDC. Colonic biopsies taken from the rectosigmoid junction of healthy controls (HC) were grown in a pIVOC system for 6 h with 108-109 Bt OMVs/ml or medium only. a) Amounts of IL-18, IL-12p70, IL-33, IL-23, IL-17a, and TNFα measured in tissue lysates are shown as mean(±SEM) values from n = 4 HC; two experimental replicates each. Statistical significance was determined used nonparametric Mann Whitney U-tests, *p<0.05. b) Absolute numbers of IL-6- (left panel) or IL-10- (right panel) expressing mDC within colonic lamina propria cells following 20 h culture with Bt (107 cfu/mL), Bt OMVs (1010/mL) or medium only. Pooled data from n=6 HC shown as mean (±SEM) numbers of cells per 10mg of tissue. Statistical significance was determined using non-parametric Kruskal-Wallis ANOVA with Dunn’s multiple comparisons test; * p<0.05.
1

## Slide 2
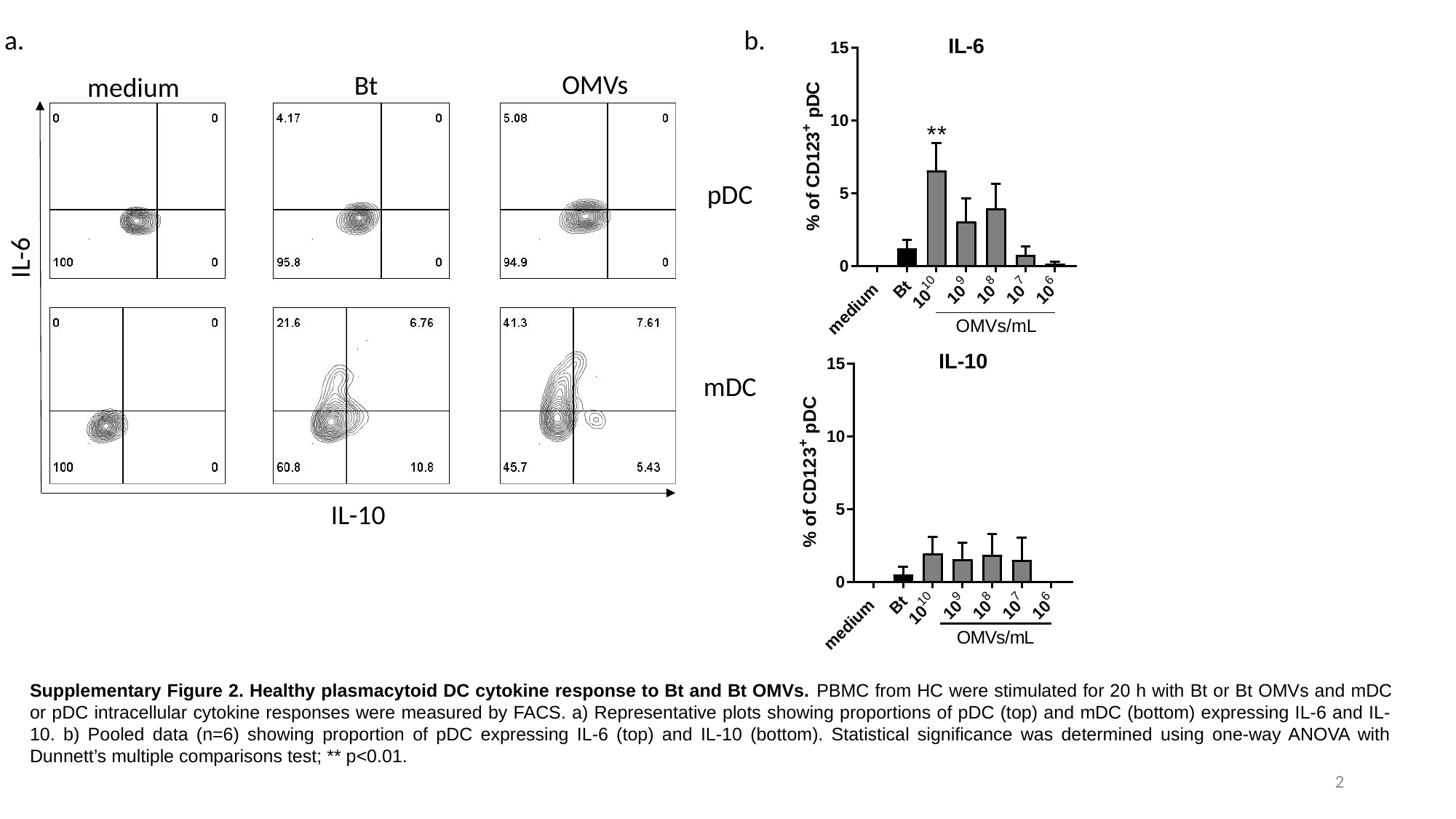

a.
b.
OMVs
Bt
medium
pDC
IL-6
mDC
IL-10
Supplementary Figure 2. Healthy plasmacytoid DC cytokine response to Bt and Bt OMVs. PBMC from HC were stimulated for 20 h with Bt or Bt OMVs and mDC or pDC intracellular cytokine responses were measured by FACS. a) Representative plots showing proportions of pDC (top) and mDC (bottom) expressing IL-6 and IL-10. b) Pooled data (n=6) showing proportion of pDC expressing IL-6 (top) and IL-10 (bottom). Statistical significance was determined using one-way ANOVA with Dunnett’s multiple comparisons test; ** p<0.01.
2

## Slide 3
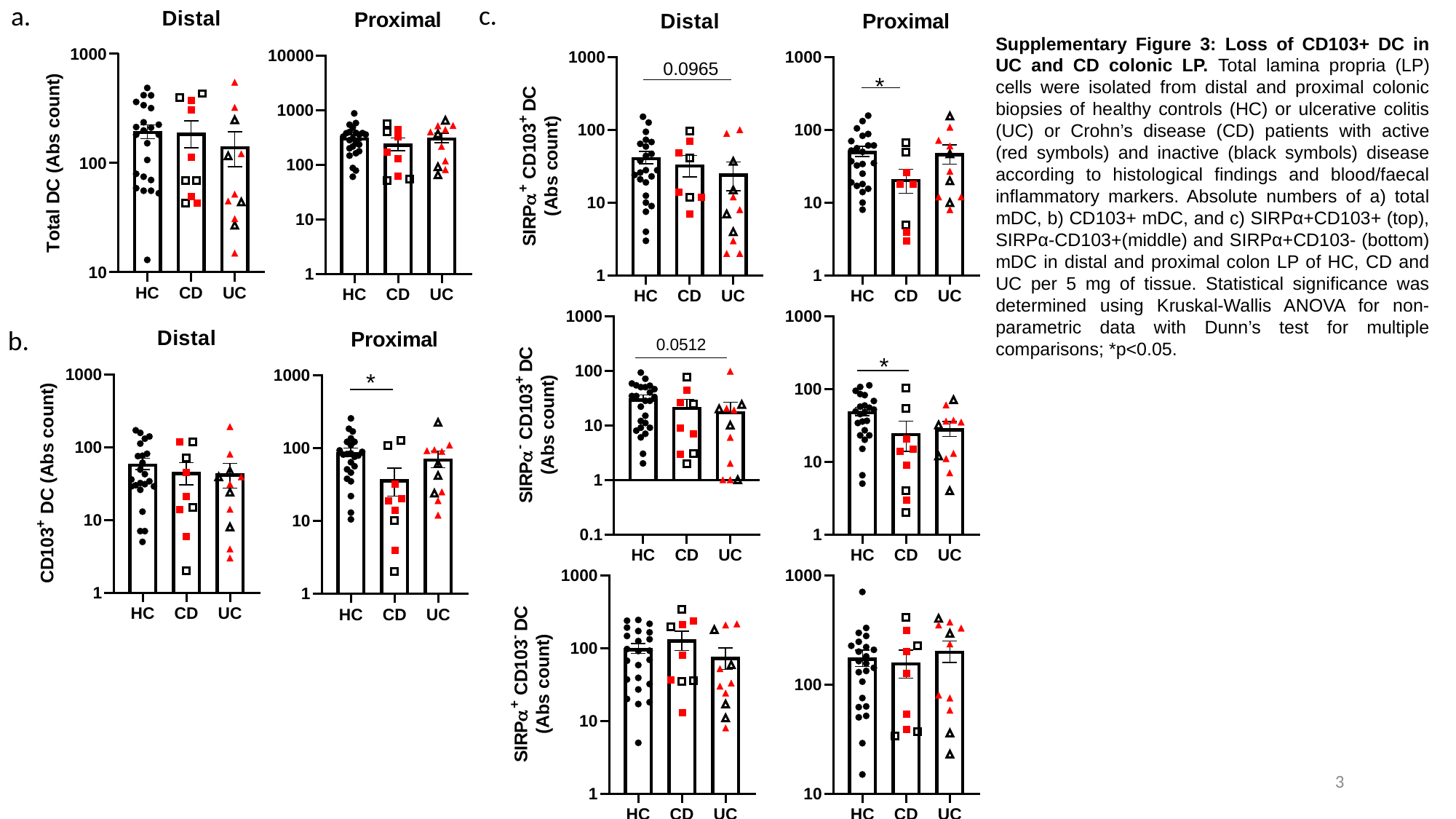

c.
a.
Supplementary Figure 3: Loss of CD103+ DC in UC and CD colonic LP. Total lamina propria (LP) cells were isolated from distal and proximal colonic biopsies of healthy controls (HC) or ulcerative colitis (UC) or Crohn’s disease (CD) patients with active (red symbols) and inactive (black symbols) disease according to histological findings and blood/faecal inflammatory markers. Absolute numbers of a) total mDC, b) CD103+ mDC, and c) SIRPα+CD103+ (top), SIRPα-CD103+(middle) and SIRPα+CD103- (bottom) mDC in distal and proximal colon LP of HC, CD and UC per 5 mg of tissue. Statistical significance was determined using Kruskal-Wallis ANOVA for non-parametric data with Dunn’s test for multiple comparisons; *p<0.05.
b.
3

## Slide 4
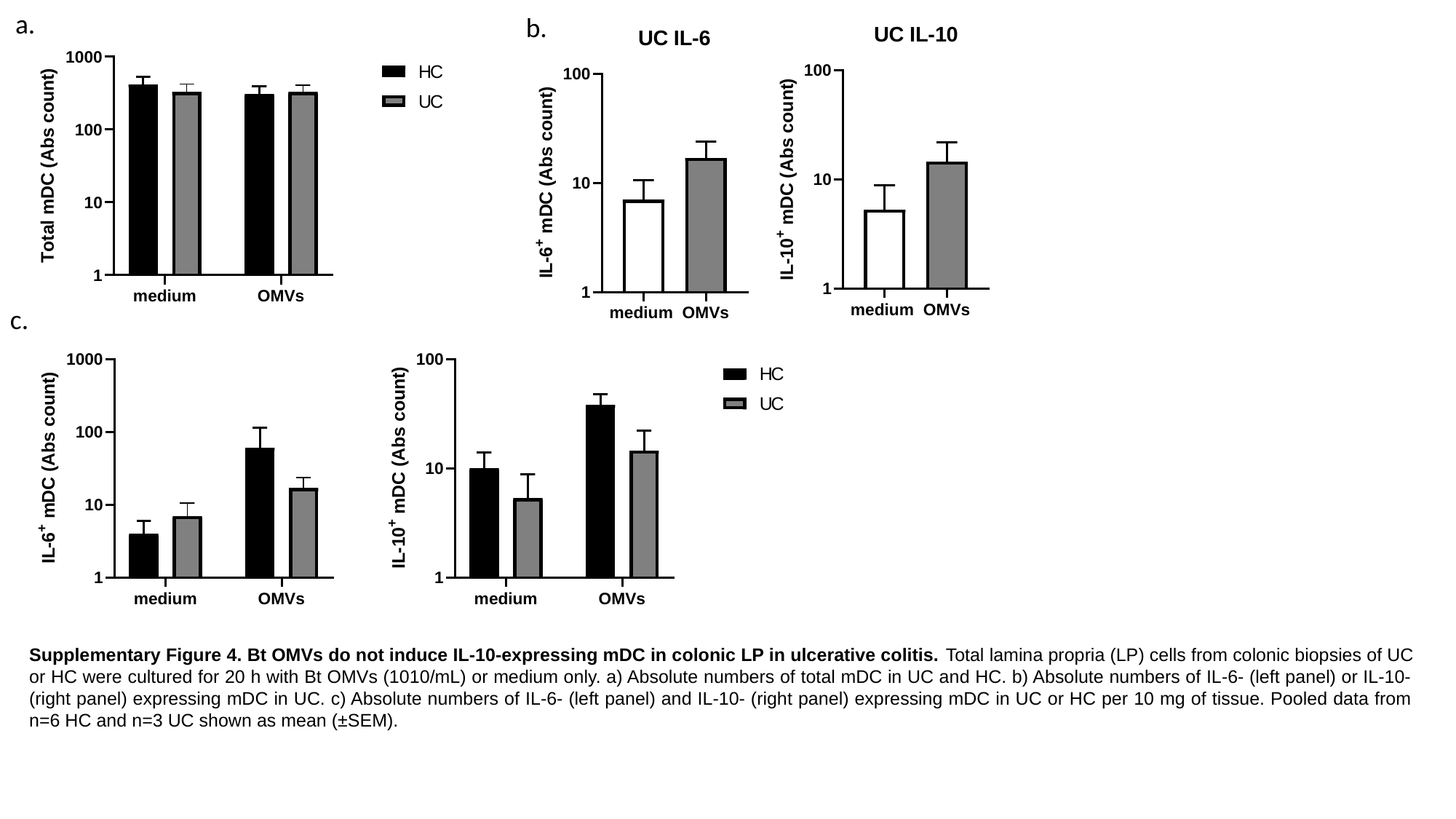

a.
b.
c.
Supplementary Figure 4. Bt OMVs do not induce IL-10-expressing mDC in colonic LP in ulcerative colitis. Total lamina propria (LP) cells from colonic biopsies of UC or HC were cultured for 20 h with Bt OMVs (1010/mL) or medium only. a) Absolute numbers of total mDC in UC and HC. b) Absolute numbers of IL-6- (left panel) or IL-10- (right panel) expressing mDC in UC. c) Absolute numbers of IL-6- (left panel) and IL-10- (right panel) expressing mDC in UC or HC per 10 mg of tissue. Pooled data from n=6 HC and n=3 UC shown as mean (±SEM).
